# Engineering Extracellular Vesicles as Delivery Systems in Therapeutic Applications

**DOI:** 10.1002/advs.202300552

**Published:** 2023-04-20

**Authors:** Liwei Wang, Di Wang, Zhaoming Ye, Jianbin Xu

**Affiliations:** ^1^ Department of Orthopedic Surgery the Second Affiliated Hospital Zhejiang University School of Medicine Hangzhou City Zhejiang Province 310009 P. R. China; ^2^ Orthopedics Research Institute of Zhejiang University Hangzhou City Zhejiang Province 310009 P. R. China; ^3^ Key Laboratory of Motor System Disease Research and Precision Therapy of Zhejiang Province Hangzhou City Zhejiang Province 310009 P. R. China; ^4^ Clinical Research Center of Motor System Disease of Zhejiang Province Hangzhou City Zhejiang Province 310009 P. R. China

**Keywords:** drug delivery, extracellular vesicles, nanocarrier, targeted therapy

## Abstract

Extracellular vesicles (EVs) are transport vesicles secreted by living cells and released into the extracellular environment. Recent studies have shown that EVs serve as “messengers” in intercellular and inter‐organismal communication, in both normal and pathological processes. EVs, as natural nanocarriers, can deliver bioactivators in therapy with their endogenous transport properties. This review article describes the engineering EVs of sources, isolation method, cargo loading, boosting approach, and adjustable targeting of EVs. Furthermore, the review summarizes the recent progress made in EV‐based delivery systems applications, including cancer, cardiovascular diseases, liver, kidney, nervous system diseases, and COVID‐19 and emphasizes the obstacles and challenges of EV‐based therapies and possible strategies.

## Graphical abstract

Extracellular vesicles (EVs) are reshaping the perspective on life science and public health. Although many EV‐based therapies have been shown to delay disease progression, the issue of EV purity and yield is a huge hurdle to overcome. In the future, how to balance the drug delivery efficiency, targeting ability, molecular activity and economic cost of EV therapeutics are also major issues worthy of research exploration.

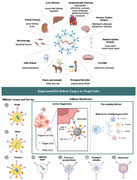

## 1 Introduction

**Figure 1:**
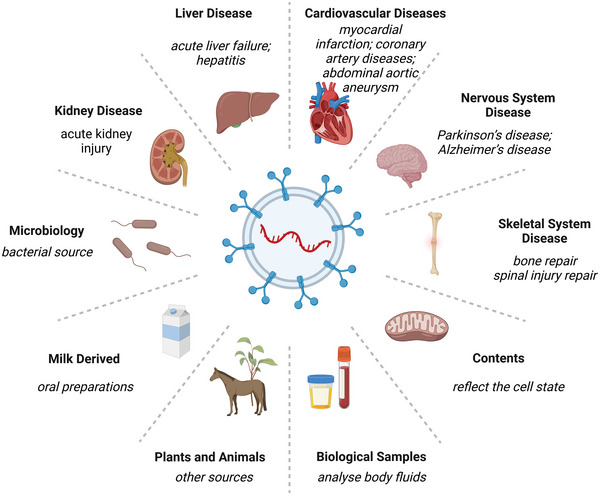
Overview of EV therapeutic application and potential sources. The analysis of EV inclusion tests based on human body fluid samples such as blood or urine helps in disease diagnosis. EV‐based therapies have been shown to be clinically effective in skeletal disorders, neurological disorders, cardiovascular disorders, and liver and kidney disorders. Nonhuman EVs based on bacteria, milk, and plants have been applied as drug carriers to achieve disease treatment.

Cells participate in the exchange of information between them through various biomolecules, including cytokines, chemokines, and metabolites. Extracellular vesicles (EVs) are lipid bilayer vesicles that are secreted from almost all cell types and represent a group of heterogeneous vesicles, which may include exosomes (secreted by endosomal pathways), microvesicles (which bud off from plasma membrane), and apoptotic vesicles (generated by cellular disintegration).^[^ ^1^ ^]^ EVs are newly recognized as the universal messengers between intercellular communications, and recent studies have shown that EVs not only carry endogenous bioactivators such as nucleic acids, proteins but also exogenously loaded therapeutics such as therapeutic RNAs, oligonucleotides, peptides, and small molecules.^[^ ^2^ ^]^ EVs are widely distributed in all body fluids, such as blood, brain effusion, saliva, amniotic fluid, and urine, and are tools for mediating information exchange between cells.^[^ ^1b^ ^]^ When the EVs bind to the recipient cells, they transmit the “cargo” into the recipient cells, thereby mediating the signal communication and substance exchange between the cells to adjust and change the function of recipient cells.^[^ ^3^ ^]^ Recent studies have shown that EVs are involved in physiological activities or pathological behavior,^[^ ^4^ ^]^ and play an important role in immune response and inflammation. Additionally, compared with normal cell‐derived EVs, “cargo” packaged in diseased cell‐derived EVs are different in abundance and variety and could be a biomarker for diagnosis.^[^ ^5^ ^]^ Particularly, EVs, as membranous vesicles widely present in organisms, have some advantages as nanocarriers for precision therapy due to their low immunogenicity and biocompatibility, among other features. Based on the unique advantages of EVs in pathophysiology, their membrane can protect its “cargo,” and the inherent or artificially modified biomacromolecules expressed on the surface of EVs can help recognize target cells or tissues.^[^ ^6^ ^]^ Therefore, EVs extensively provide a source of delivery vehicles and even treatment means (**Figure** **1**).

Most of the currently popular drug delivery systems rely on the use of synthetic nanocarriers, such as polymeric liposomes and metal nanoparticles, which can better improve the bioavailability and enhance the stability of the drug.^[^ ^7^ ^]^ 2018 saw the first FDA approval of the lipid nanoparticle (LNP)‐based siRNA drug Patisiran (trade name Onpattro) for the treatment of hereditary thyroxine transport protein‐mediated amyloidosis (hTTR).^[^ ^8^ ^]^ Additionally, in recent years, LNP‐based mRNA vaccines have achieved important results in the fight against COVID‐19.^[^ ^9^ ^]^ In general, synthetic nanocarriers are more efficient in encapsulating drugs and have low toxicity and low immunogenicity. However, low uptake and low targeting due to low biocompatibility are also important reasons limiting their application.^[^ ^7^ ^]^ EVs derived from cell therapy have attracted the attention of researchers because of their superior biocompatibility, low immunogenicity, modifiability, targeting modifiability, and ease of crossing physiological barriers, and have become a promising drug delivery vehicle.

## 2 EV Biology

EV is a highly conserved intercellular communication and nanocarrier system that originated in the endosomal system and whose formation is associated with multivesicular bodies (MVBs) and intraluminal vesicles (ILVs).^[^ ^1^, ^10^ ^]^ The ILVs are generated from the inward budding of the endosomal membrane during the maturation of MVBs. Ultimately, ILVs are secreted as EVs with a diameter range from 40 to 160 nm when MVBs fuse with the endosomal membrane.^[^ ^11^ ^]^ EVs were first identified in the reticulocytes of sheep and are involved in removing unwanted proteins.^[^ ^12^ ^]^ Subsequent studies have shown that EVs play an important regulatory role in the immune system.^[^ ^13^ ^]^ During the early 21st century, researchers discovered that EVs could transmit biological information^[^ ^14^ ^]^ between cells depending on RNA,^[^ ^15^ ^]^ lipids, and proteins.^[^ ^16^ ^]^ Recently, the significance of EVs in endo‐environmental homeostasis,^[^ ^17^ ^]^ inflammation regulation, and tumor progression^[^ ^18^ ^]^ has been elucidated. The biological properties of EVs have been elucidated in many studies,^[^ ^19^ ^]^ and will not be discussed in detail in this review. In this review, we will focus on the recent years of EVs as carriers for the delivery of endogenous or exogenous cargoes for treating relevant diseases, and discuss the latest relevant methods for EV isolation, loading, and storage, and provide thoughts on their breakthroughs in the medical field.

## 3 EVs as Potential Delivery Vehicles

**Figure 2:**
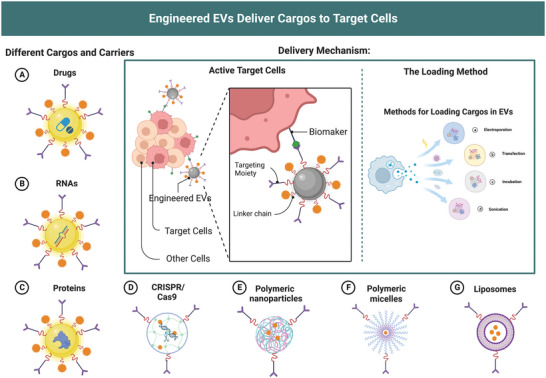
EVs loading strategies and delivery mechanism. Engineered EVs can recognize target cells via biomarkers consisting of EV‐sorting domains fused with selective proteins and loading cargo by electroporation, transfection, incubation, and sonication. EVs are loadable with exogenous therapeutic cargos, including A) drugs, B) RNAs, C) proteins, and D) CRISPR/Cas9, similar to mainstream drug delivery systems like E) polymeric nanoparticles, F) polymeric micelles, and G) liposomes.

EVs are stable in biofluids and organisms, and they can distribute over short and long distances, even penetrating the biological barrier.^[^ ^20^ ^]^ EVs are unique in protecting and delivering their internal cargos to target cells through ligand–receptor interactions. Previous studies have shown that proteins on EV surfaces facilitate cargo delivery and increase half‐lives in circulation by promoting membrane fusion with the target cell and suppressing CD47‐mediated phagocytic clearance, enhancing the pharmacological properties of EVs.^[^ ^21^ ^]^ EV uptake depends on surface ligands like HSPGs or recipient cell surface receptors like SR‐B1. Recent studies have found that EVs are predisposed to specific organs, based on which we can load cargo on them and deliver them to targeted recipient cells. Also, other issues need to be considered; for example, the size of engineered EVs should be small enough to evade reticuloendothelial system (RES) uptake and large enough to avoid rapid renal clearance. As nanometer‐sized particles, EVs can easily be transported through body fluid and biological barriers.^[^ ^22^ ^]^ Assuming that this specific approach can be precisely controlled with high efficiency, EVs will be an effective tool for transferring therapeutic elements.^[^ ^23^ ^]^ Several biological molecules like miRNAs, siRNAs, and complex recombinant proteins or molecules are difficult to deliver intracellularly without carriers, but engineered EVs can load specific cargo into target cells and induce genetic modification through endocytosis (**Figure** **2**).

**Figure 3:**
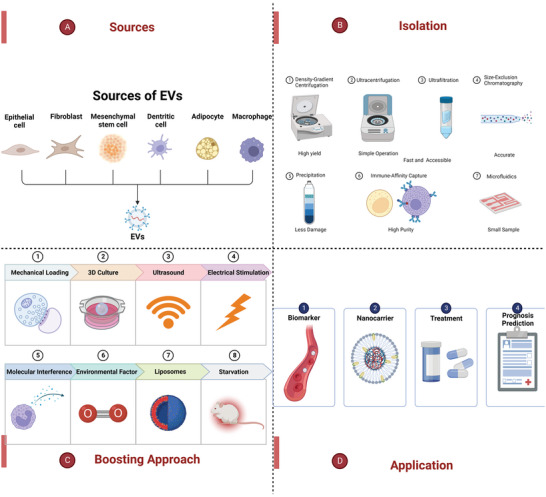
An overview of sources, isolation, boosting approach, and the application of EVs. A) EVs can be secreted by various cells with different biological functions. B) The methods of isolating EVs, including density‐gradient centrifugation, ultracentrifugation, ultrafiltration, size‐exclusion chromatography, precipitation, microfluidics, and immune‐affinity capture. C) The methods for boosting EV secretion, including physical signals, molecular interference, environmental factors and external inducers. D) The application of EVs as biomarkers or nanocarriers for disease diagnosis, prognosis, or treatment.

When developing EV therapies, the primary consideration is the selection of donor cells for EVs (**Figure** **3**A). Theoretically, almost all living cells can secrete EVs, but if EVs are to be used in therapy, the basic thing is to ensure their safety. However, if EVs are to be used therapeutically, it is fundamental to ensure their safety, according to Good Manufacture Practice (GMP) principles, to ensure that EVs secreted by donor cells do not cause serious adverse effects such as proinflammatory, teratogenic, and carcinogenic effects in humans. The choice should then be made in relation to the therapeutic target, as EVs from different sources generally retain the characteristics of the donor cells. For example, MSCs‐EVs can induce angiogenesis and modulate immune signaling, as well as strong tissue repair;^[^ ^24^ ^]^ EVs from immune cells contain major histocompatibility complex (MHC), which can induce or inhibit specific immune responses; tumor‐derived EVs show an extremely strong homing ability but may also lead to tumor progression and metastasis; and platelet‐derived EVs have a natural affinity to damaged sites in the vessel wall.^[^ ^25^ ^]^ Therefore, the source of their donor cells is also worthy of primary consideration when employing EV therapy.

Currently, immature DC‐derived EVs,^[^ ^26^ ^]^ mesenchymal stem cells‐derived EVs,^[^ ^27^ ^]^ or HEK293‐derived EVs serve as nanocarriers for drug delivery.^[^ ^28^ ^]^ Among them, immature DC‐derived EVs have the lowest toxicity and weaker ability to induce an immune response. However, a disadvantage is that the amount of collectible EVs is relatively low. Compared with tumor‐derived EVs,^[^ ^29^ ^]^ DC‐derived EVs can induce more efficient anti‐tumor immunity.^[^ ^30^ ^]^ The amount of EVs secreted by mesenchymal stem cells (MSCs) and HEK293 cells is large. MSCs come from various sources and show excellent stability and sustainability in human plasma at −20 °C. Human bone marrow MHCs incubated with drugs have anti‐tumor effects.^[^ ^31^ ^]^ Also, cell immortalization does not affect the quantity and quality of EV production, ensuring the possibility of a continuous supply of EVs. But it has been found that tumor‐derived EVs may induce malignant changes in targeted cells because of the related miRNAs.^[^ ^32^ ^]^ Therefore, we believe that their donor cells should have the following characteristics: ① The secreted EVs are simple or almost free of impurities ② The cell line is stable, easy to obtain, and easy to culture ③ The cell secretes a large amount of EVs (has a high EV production capacity) ④ The cell is easy to handle and transfect ⑤ The EVs secreted by the cell do not show any toxicity or immunogenicity in vivo and in vitro.

The currently proposed EVs of nonhuman origin include prokaryotes (i.e., bacteria),^[^ ^33^ ^]^ protists (various algae), milk,^[^ ^34^ ^]^ various plants,^[^ ^35^ ^]^ and other animals.^[^ ^36^ ^]^ Compared to human‐derived EVs, nonhuman‐derived EVs are more easily adapted in production, loading and target modification,^[^ ^37^ ^]^ and their potential application areas are not narrower than those of human‐derived EVs. For example, meningococcal group B‐derived EVs have been approved for vaccine manufacture in Cuba, New Zealand, and Brazil.^[^ ^38^ ^]^ In contrast, EV isolated from ginger extract has been shown to activate antioxidant gene expression to protect mice from alcohol‐related liver injury^[^ ^39^ ^]^ and to target the colon after oral administration, with 6‐gingerol and 6‐shogaol reducing acute colitis and enhancing intestinal repair without altering cell viability after 24 h of treatment.^[^ ^40^ ^]^ Milk‐derived EVs may be associated with the MAPK signaling pathway, while Wu et al. found that oral administration of milk‐derived EV‐loaded insulin produced superior and longer‐lasting hypoglycemic effects in type I diabetic rats compared to subcutaneously injected insulin,^[^ ^41^ ^]^ which provides novel insight into oral insulin administration. Milk‐derived EVs can exist under degradation conditions in the strongly acidic conditions of gastric juice and in the presence of digestive enzymes in the intestine,^[^ ^42^ ^]^ as oral drugs, significantly reducing the cost and inconvenience associated with intravenous therapy. Additionally, milk‐derived EVs promote ulcer wound healing in patients with diabetes^[^ ^43^ ^]^ and can also be used to load nucleic acid drugs like siRNA,^[^ ^44^ ^]^ and miRNA.^[^ ^34^ ^]^ Although nonhuman EVs are also increasingly studied, there are still some critical issues that need to be addressed. Nonhuman‐derived EVs may be immunogenic or sensitizing when repeatedly administered over a long period of time. Moreover, the chemical characteristics of nonhuman EVs are different from those of human EVs, and caution may be needed in conducting relevant analyses and studies with experimental biases brought about by their different markers or active molecules from human EVs. Also, data comparing their effects with those of mammalian‐derived EVs are lacking.

## 4 EVs as Delivery Vehicles

### 4.1 Advantages of EVs as Nanocarriers

Currently, the mainstream drug delivery systems are liposomes and polymeric nanoparticles.^[^ ^7^ ^]^ Liposomes are phospholipid membrane‐synthesized vesicles of various sizes and forms that can transport any hydrophilic or hydrophobic molecule. Polymeric nanoparticles^[^ ^45^ ^]^ comprise biocompatible and degradable materials, such as natural polymer collagen and synthetic polymer polyacrylates. They facilitate drug transportation and increase drug half‐life to improve the pharmacological properties of drugs. Compared with the mainstream drug delivery systems, EVs have a relatively small molecular structure, the properties of natural molecular transport, and good biocompatibility.^[^ ^46^ ^]^ There are two basic principles for selecting a pharmaceutical nanocarrier: protecting the contained drugs from inactivation in the environment in vivo and releasing the contained drugs without inducing an immune response to the nanocarriers. EVs have significant advantages for drug delivery over current nanocarriers like liposome‐based or polymer‐based nanocarriers (**Table** **1**). First, EVs come from natural cells, and their inclusions can transfer and alter the function of recipient cells. Second, compared with the lower packaging efficiency of liposomes for hydrophilic molecules, inclusion is limited in the delivery of nucleic acid. EVs could effectively attract nucleic acids and significantly improve packaging efficiency. Third, EVs are derived from special cells, including immature DC cells or MSCs. The special molecules on the surface of EVs can avoid interaction with opsonin, antibodies, and coagulation factors, thus avoiding the immune response in vivo. Additionally, compared with liposome‐based or polymer‐based nanocarriers, EVs have higher stability in body fluids such as stability in blood. Lastly, through the inherent or modifiable molecules on the surface of EVs, it can have a targeting effect that selectivity binds to the recipient cells. In summary, these studies highlight that EVs loaded with cargo are promising multifunctional nanotherapeutics to relieve diseases symptoms. Of course, there are studies that have broken away from the original inherent view by combining liposomes with EVs, incorporating both advantages to prepare drug delivery systems with better stability, drug loading capacity, and slow‐releasable drugs.^[^ ^47^ ^]^ The future trend will be to combine EV delivery systems with intelligent technologies to achieve real‐time detection and control of drugs, thus realizing personalized drug therapy and precision medicine. In addition, with the development of gene therapy and noncellular therapy, the application of EVs will also become an integral part of these therapeutic modalities. Therefore, EV‐based drug delivery systems have a very broad application prospects in the future medical field.

**Table 1:** The advantages of EVs as delivery systems

Characteristic	EVs	Liposome	Polymer
Size	40–100 nm	25–1000 nm	50–1000 nm
Source	Natural cells	Liposome synthesis	Chemical synthesis
Structure	Phospholipid bilayer	Double‐layer membrane	Polymerization
Composition	Proteins, lipids, and nucleic acids	Fat‐soluble molecules	Polymers
Toxicity	Low	Low	High
Stability	High	Low	High
Efficiency	High	Middle	High
Immunogenicity	Low	Middle	High
Targeting capacity	Inherent/modifiable	Modifiable	Modifiable
Application	Not available	Available	Available
Limitation	Low yield and absence of clinical evaluations	Low stability and Limitation of encapsulation	Potential toxicity and Potential immunogenicity
Recommendation	Establishing standards and optimizing processes	Compound liposome	Improvement of materials and methods

### 4.2 The Method of EVs Isolation and Engineering

According to the typical structural characteristics of EVs,^[^ ^48^ ^]^ such as density, size, and surface markers, the method of isolating EVs can be divided into seven: density‐gradient centrifugation (DGC), ultracentrifugation (UC), ultrafiltration (UF), size‐exclusion chromatography (SEC), precipitation, microfluidics and immune‐affinity capture (Figure 3B).^[^ ^49^ ^]^ Each method has its superiority and deficiencies. However, none can completely isolate pure EVs. The selected method affects the amount, purity, and physicochemical properties of EVs obtained. Parameters including efficiency, purity, accessibility, cost, and reliability should be considered when choosing the isolation method. DGC can isolate particles with a high yield but low purity. Ultracentrifugation, a gold standard method for EV isolation, can distinguish EVs based on their size, density, and shape, and it is easy to operate with large sample capacity and can isolate high concentrations of EVs.^[^ ^50^ ^]^ The disadvantages are that ultracentrifugation is time‐consuming, has low‐recovery, has high equipment requirements and has high speed, which may damage EVs, decreasing the biological activity. The principle of ultrafiltration is to separate EVs based on the particle size difference. It is a process that removes impurities with membrane filters and pressure; here, the products will be of low purity and shattered.^[^ ^51^ ^]^ But UF is faster and more accessible than ultracentrifugation and can be used on a large scale.^[^ ^52^ ^]^ SEC, a column separation method, also exploits size differences and can completely and accurately isolate the EVs without damaging them, although it is time‐consuming.^[^ ^53^ ^]^ Precipitation separates EVs by changing their solubility. It is suitable for large sample volumes and causes little damage to EVs.^[^ ^54^ ^]^ However, this strategy is time‐consuming and has low purity; free protein contamination is the main concern.^[^ ^55^ ^]^ As for microfluidics, a new technique that uses both physical and biochemical characteristics of EVs to isolate them, is a cheap and time‐saving approach to obtain EVs with a small amount of sample, but it inevitably has low sensitivity, and is unsuitable for large‐scale sample.^[^ ^56^ ^]^ The final strategy is immune‐affinity capture, a technique based on the interaction between antigens and antibodies like CD9, CD63, CD81, and Alix. Its strengths are high purity and defined subtype, while the problems are low yield, high cost, and risk of antigen blockade, or even loss of function; it also requires extra separation and purification steps after binding to antibodies.^[^ ^57^ ^]^ Each method has its drawbacks. An ideal isolation method is supposed to maintain vesicle integrity and should be simple, fast, and low‐cost with both high sensitivity and specificity. According to statistics, the use of ultracentrifugation accounts for approximately 56% of EV‐based studies,^[^ ^58^ ^]^ which is the most widely used technique. At the same time, ultracentrifugation is also the easiest to operate and the most technically developed, so it is expected to be the most promising technology for industrial production.^[^ ^59^ ^]^ Under current conditions, efficiency and EV losses can be minimized by optimizing centrifugation conditions such as pretreatment of samples, adjusting centrifugation speed and time, and combining other purification techniques according to downstream needs. Furthermore, in addition to the currently favored microfluidic technology, we believe that the application of nanopore electrophoresis or nanopore filter membrane technology to the separation and purification of EVs may also be very feasible. Additionally, standard purification protocols and safety profiles must be followed to reduce the biological side effects of contaminants, so further research is needed to avoid these problems and to prepare the technique for future EVs research.

However, the yield of isolated EVs still has limitations, and it is necessary to increase exosome production. Presently, some biological methods have been applied to increase exosome production, including physical signals, molecular interference, environmental factors, and external inducers. The goal is to isolate and obtain sufficient EVs for clinical applications such as diagnosis and treatment (Figure 3C,D).

The important technique in EV engineering is to make EVs carrying target molecules by certain means while also allowing for targeted modifications that can be used for relevant therapeutic purposes. For delivery purposes, there are different loading strategies, which are called differently in diverse articles, but the principles are the same. The loading of cargos can be divided into endogenous loading and exogenous loading.^[^ ^60^ ^]^ Endogenous loading is based on the modification of donor cells of EVs so that the secreted EVs carry the target molecules. Exogenous loading, on the other hand, is where EVs are first isolated and purified and then cargo molecules are loaded directly onto the EV membrane surface or into the membrane. Endogenous loading does not damage EV membranes, and the most commonly used methods are coincubation (here coincubation refers to cargo molecules and donor cells) and transfection. However, the limitation of endogenous loading is the inability to control the amount of drug loaded on EVs. Exogenous loading allows for higher loading efficiency with the help of physical or chemical methods, such as direct loading of target molecules into EVs by electroporation, ultrasound, extrusion, freeze‐thaw cycles, and chemical drugs. This method is suitable for mass production, but has limitations due to disruption of the EV membrane, causing EV aggregation or cleavage. Exogenous loading can also be performed by coincubation (here coincubation means cargo molecules and EVs), which can be enhanced by applying external forces such as shaking or stirring. It is particularly suitable for hydrophobic drugs, but has the disadvantage of low loading efficiency. Currently, emerging techniques for exogenous loading, such as click chemistry, bind cargos to EVs covalently, whose release depends on EV degradation. In addition, one study explored fusing Lamp2a with the TAT peptide of EVs and exchanging the loop of the target miRNA (premiR‐199a loop) with the TAR RNA loop, thus allowing the modified miRNA to recognize EVs through TAT‐TAR interactions, increasing the loading by 65‐fold. Compared with endogenous loading, exogenous loading is less difficult to experiment and has a shorter experimental cycle. However, as we know, each loading method has its limitations, there is no uniform standard loading method, and researchers can select the appropriate method based on specific downstream experiments.

Targeting of EVs is another important topic that utilizes tissue‐specific peptides or ligands to modify EVs by means of membrane fusion, chemical modification, and genetic engineering.^[^ ^61^ ^]^ Among them, membrane fusion is the fusion of the phospholipid bilayer of EV membranes with exogenous lipid membrane structures with targeted functions by filtration, extrusion, or freeze‒thaw cycles, and its simplicity of operation technique is the biggest advantage. Lysosomal‐associated membrane protein 2 (Lamp2) is expressed abundantly on EVs; therefore, they are extensively chosen as fusion proteins to steer the engineered EVs toward target cells.^[^ ^62^ ^]^ Besides, lactadhesin and platelet‐derived growth factor receptors are also widely used transmembrane proteins in EV targeting. For example, Li et al.^[^ ^63^ ^]^ used extrusion to fuse platelet membranes with EV membranes, and the obtained products, P‐EVs, not only retained the original functions of EVs, but also inherited the ability of platelets to target damaged blood vessels, which demonstrated good myocardial repair ability in mouse models of myocardial ischemia‒reperfusion. Chemical modification can be divided into covalent and noncovalent methods. The covalent method is also known as binding the targeting part to the EV membrane through covalent bonding, while the noncovalent method is the binding of the targeting part to the EV membrane under the action of noncovalent bonds such as classical interactions, electrostatic interactions, and receptor–ligand interactions. As we understand, noncovalent modification has a small bond energy and weak binding strength compared to covalent modification. Additionally, the improvement of targeting ability can be addressed by means of genetic engineering, which involves changing the core of the particle and is not limited to the surface of the EV membrane compared to chemical modifications. For example, Cheng et al.^[^ ^64^ ^]^ enabled donor cells to secrete EVs that could express two different types of antibodies by transfecting Expi293F cells (a suspension‐adapted HEK293 cell line), which could target both CD3 on the surface of T cells and triple‐negative breast cancer cells expressing EGFR (TNBC). Both in vivo and in vitro experiments have shown that engineered EVs specifically kill TNBCs when induced by the immune system. Although targeted modifications can cause EVs to specifically accumulate at the target site, their stability and effects remain to be elucidated and improved.

In engineering EVs, we believe that the issue of the activity of the therapeutic molecules is of most concern, and increasing the loading without improving the activity of the loaded molecules may not be a practical solution to the problem. At the same time, excessive loading of EVs may interfere with the uptake mechanism of EVs, as the loadings themselves may carry electrical charge and thus can affect the surface charge of EVs. Recently, in Didiot's study,^[^ ^65^ ^]^ binding siRNA to cholesterol to form hydrophobically modified siRNA and then coincubating it can significantly increase the loading and in vivo activity of siRNA without changing the distribution and integrity of EVs, which provides a new idea to improve the efficiency of endogenous loading. In addition, one study explored^[^ ^66^ ^]^ the EV anchoring peptide CP05 obtained by the phage display technique, which not only specifically binds the surface marker CD63 of EVs, but also binds nucleic acid molecules, and thus can effectively modify, load, or capture EVs. These studies have opened a new avenue for EV therapeutic applications (**Table** **2**).

**Table 2:** The application of EVs as delivery systems

Sources of EVs	Types of drug	Recipient cells	Routes of administration	Mechanisms of action	Refs.
HEK293T cells	Doxorubicin	Thyroid cancer cells	Tail vein injection	Dox and I^131^ target to tumor via integrin *α*v*β*3	[67]
Blood	Dox and miR‐21i	Glioblastoma	Tail vein injection	Dox and miR‐21i target to tumor via superparamagnetic nanoparticle cluster	[68]
Blood	cPLA2 and siRNA	Glioblastoma	Intravenously administered	cPLA2 knockdown and metformin treatment	[69]
HEK293T cells	Doxorubicin	Glioblastoma	Tail vein injection	Dox target tumor via angiopep‐2 and trans‐activator	[70]
Macrophage cells	Curcumin and albumin	Inflammatory cells	Apply with fingers for ten minutes	Downregulate NF‐*κβ* and target via albumin	[71]
MSCs	siRNA	Brain cells	Tail vein injection	siRNA silencing Htt gene of brain via G58 peptide	[72]
HEK293T cells	siRNA and cholesterol	Brain cells	−	−	[73]
Chondrocytes	miRNA‐140	Osteoarthritis cells	Injected intraarticularly	miRNA‐140 target chondrocytes via CAP	[74]
Tendon stem cells	miRNA‐144‐3p	Tenocytes	Tendon injection	miRNA‐144‐3p download ARID1A	[75]
HEK293T cells	miRNA‐124	NK cells	−	Inhibit M2 microglial polarization	[76]
HCT‐116 cells	5‐Fu and miRNA‐21i	Colorectal cancer cells	Tail vein injection	miR‐21i increase the sensitivity of 5‐FU by rescuing the expression of hMSH2 and PTEN	[77]
HepG2 cells	ASOs G3139	Bcl‐2	−	Increase the delivery of G3139 with downregulation of antiapoptotic Bcl‐2	[78]
HEK 293 cells	GFP	Target receptor cells	−	−	[79]
LX‐2 cells	Cas9 RNP	p53	Tail vein injection	Target p53 to upregulate PUMA, CcnE1, and KAT5	[80]
Murine C2C12 cells	Morpholino oligomers	Muscle	Tail vein injection	Target muscle via CP05 increasing dystrophin protein	[66]
HeLa cells	CRISPR/Cas9	HBV	−	CRISPR/Cas9 cut HBV DNA or HPV DNA specifically	[81]
KPC689 cells	CRISPR/Cas9	Pancreatic cancer cells	Tail vein injection	CRISPR/Cas9 target the mutant KrasG12D oncogenic allele	[82]

### 4.3 Delivery of Cargos

#### 4.3.1 Delivery of Small Molecule

Currently, chemotherapeutic drugs are hydrophobic and have poor pharmacokinetic properties.^[^ ^83^ ^]^ This makes the delivery of small molecule‐drugs challenging. Meanwhile, the nonspecific distribution of drugs in the body leads to serious side effects and systemic toxicity. Hence, the development of drug delivery systems that can target tumor sites is a realistically urgent need. The use of EVs for drug loading can improve drug solubility and reduce their toxicity, while the use of ligands grafted on the surface of engineered EVs can improve the targeting of drug delivery and efficacy. Wang et al. constructed an EV‐targeting delivery system by fusing Lamp2b from EVs produced by HEK‐293T cells to an integrin‐specific iRGD peptide fragment and a tyrosine fragment.^[^ ^67^ ^]^ With this engineered EV loaded with doxorubicin (Dox) and labeled with radioactive I^131^ using the chloramine‐T method, the system showed good targeting ability and efficient Dox‐delivery efficiency in confocal imaging and flow cytometry. In a mouse 8505C xenograft model, the EV‐targeting delivery system showed significant tumor suppression by intravenous injection without significant side effects. Additionally, Zhan et al. embedded Dox and cholesterol‐modified miRNA21 inhibitor (miR‐21i) into blood‐derived EV lipid bilayers and observed significant tumor growth inhibition in tumor‐bearing mice,^[^ ^68^ ^]^ showing that they retained their original properties and possessed tumor‐targeting and efficient transfection properties. Glioma is a primary malignant brain tumor in adults with poor prognosis, and the existence of the blood‐brain barrier (BBB) is the main challenge of targeted therapy for glioma. Studies have shown that the EV‐mediated cPLA2 (cytoplasmic phospholipase A2) siRNA/metformin combined strategy could cross BBB and accumulate at the glioblastoma site to modulate energy metabolism.^[^ ^69^ ^]^ After 21 d, found that the treatment group therapy effect is 6.7 times higher than the PBS group. Low‐density lipoprotein receptor‐related protein‐1 (LRP1) mediates multiple ligands across the BBB and is abundantly expressed in gliomas, while Angiopep‐2 (Ang) is a targeting peptide with a high affinity for LRP1 and trans‐activator of transcription (TAT) is an efficient cell‐penetrating peptide. Zhu et al. modified EVs through the synergistic effect of Ang and TAT, a drug delivery system (Ang/TAT‐EXOs‐Dox) with Ang‐mediated glioma targeting ability and TAT‐mediated efficient cell membrane penetration ability.^[^ ^70^ ^]^ Besides, Yerneni et al. encapsulated curcumin and albumin into EVs using mild sonication, and integrated the curcumin‐albumin‐EVs (CA‐EVs) into tip‐loaded dissolvable microneedle arrays (dMNAs). Curcumin in CA‐EVs exhibited five‐fold higher stability than curcumin alone or curcumin‐EVs without albumin.^[^ ^71^ ^]^ The natural molecular structure of EVs provides a basis for the transport and uptake of small molecule drugs. EVs reduce the toxicity of drugs, but also improve the utilization efficiency of drugs. It has natural advantages as nanocarriers for small‐molecule drug delivery compared with traditional delivery systems.^[^ ^84^ ^]^

#### 4.3.2 Delivery of Nucleic Acid

RNA interference (RNAi) is the specific inhibition of gene expression after transcription to achieve gene silencing, and small interfering RNA (siRNA) performs this process.^[^ ^85^ ^]^ However, these siRNAs are unstable and degrade rapidly in circulation. EVs can protect siRNAs and deliver them to targeted cells.^[^ ^86^ ^]^ Dar et al. identified many free GAPDH binding sites on the phospholipid bilayer of EVs. GAPDH can bind to EVs through the phosphatidylserine (PS)‐bound G58 structural domain, thus promoting EV aggregation. Therefore, they used this binding site to construct a siRNA delivery system with high loading (about 500–700 siRNAs per EV) and protection efficiency. They also found that intravenous administration in mouse models of Huntington's disease resulted in the silencing of disease‐related genes in different regions of the brain, slowing down disease progression.^[^ ^72^ ^]^ Also, it has been found that EV loading of cholesterol‐binding siRNA (CC‐siRNA) can effectively increase siRNA loading and has also been validated in mouse models of Huntington's disease.^[^ ^73^ ^]^ To combine the advantages of biological and synthetic drug delivery, some researchers have hybridized liposomes with EVs using film hydration and extrusion.^[^ ^87^ ^]^ The hybrids can reduce liposome toxicity and retain the intrinsic functions of parent cell EVs, such as the ability of cardiac progenitor cell (CPC) EVs to stimulate cell proliferation and migration. They can also be loaded with siRNA to retain gene silencing. Thus, hybrid nanoparticles can serve as the best of both worlds for a therapeutic approach.

miRNAs are a class of non‐coding endogenous RNAs primarily used to regulate post‐transcriptional gene expression.^[^ ^88^ ^]^ miRNA inhibits gene expression and degrades the mRNA by binding to untranslated regions of the mRNA (UTRs).^[^ ^89^ ^]^ Synthesized miRNAs have a short half‐life in circulation and can be improved using chemical modification and, more extensively, by loading into EVs. Drug entry into chondrocytes through the outer matrix of the avascular component of cartilage remains a major challenge in osteoarthritis treatment. microRNA‐140, a miRNA that plays a protective role in chondrocytes, has been loaded with an engineered EV fusing Lamp2b with chondrocyte affinity peptide. The delivery system can target the deep cartilage regions of the joints, inhibit cartilage‐degrading proteases, and alleviate osteoarthritis progression in a rat model.^[^ ^74^ ^]^ Furthermore, tendon stem cell‐derived EVs loaded with miR‐144‐3p can promote tendon repair by promoting tenocyte proliferation and migration.^[^ ^75^ ^]^ Besides, EVs loaded with mi‐R124 can regulate the STAT3 signaling pathway in glioblastoma (GBM) and microglia, and recruit NK cells into tumors, exhibiting efficient anti‐tumor effects.^[^ ^76^ ^]^ It has also been found that engineered EVs loaded with 5‐Fu and miR‐21i effectively downregulated miR‐21 expression in the colorectal cancer cell line HCT‐1165FR, reducing tumor proliferation, increasing apoptosis and rescuing the expression of PTEN and hMSH2. Drug resistance was effectively reversed, and 5‐Fu cytotoxicity was enhanced compared with 5‐Fu or mi‐R21i alone in a mouse model.^[^ ^77^ ^]^

mRNA is a class of single‐stranded ribonucleic acids that is transcribed from a strand of DNA as a template and carries genetic information that can guide protein synthesis. It also has unfavorable pharmacokinetic properties due to its susceptibility to degradation, which increases the need for an effective delivery system. An excellent delivery system protects mRNA from degradation by nucleases in vivo and can induce uptake by target cells. Previously, it was demonstrated that lipid nanoparticles can efficiently and specifically deliver mRNA to the pancreas.^[^ ^90^ ^]^ Nawaz et al. further found that when delivering VEGF‐A mRNA via LNPs, part of the internalized mRNA continued to function through EV secretion, and at the same VEGF‐A protein content, different cell‐secreted EVs had different degrees of effect.^[^ ^91^ ^]^ In general, LNPs and EVs can be interconnected, and after the delivery of therapeutic molecules by LNPs, the EVs secreted by cells contain more molecules with similar biological functions in addition to therapeutic molecules, so EVs can be regarded as the functional expansion of LNPs. Therefore, extending the use of the better performance of EVs to guard mRNAs as their loading vehicles, the Kojima‐designed EXOtic for in situ production of EVs was observed to indeed produce enough therapeutic EVs to rescue Parkinson's‐associated neurotoxicity and neuroinflammation after loading relevant therapeutic mRNAs.^[^ ^92^ ^]^

Bcl‐2 overexpression can cause chemoresistance, and antisense oligonucleotides (ASOs) can target Bcl‐2. However, nucleotides are prone to rapid degradation and the negative charge they carry makes them difficult to cross cell membranes. Coupling cell‐penetrating peptides to the surface of HepG2 cell‐derived EVs improves the penetration ability of EVs and allows better loading of ASOs on EVs. G3139 is an octadecameric ASO, and inserting it into the EV membrane or adsorbing it to peptides effectively prevents its degradation and increases ASOs transport with greater penetration ability.^[^ ^78^ ^]^ By investigating its cellular uptake mechanism, the codelivery system was found to significantly increase the cellular delivery of G3139 by effectively down‐regulating the antiapoptotic gene Bcl‐2, providing more possibilities for the delivery of nucleic acid drugs.

#### 4.3.3 Delivery of Proteins

EVs have been intensively explored for the therapeutic delivery of proteins, and the modification of EVs by protein cargos is usually achieved through cellular engineering. Plasmids encode and overexpress target proteins, which are then naturally sorted into EVs or fused to membrane proteins during EV generation. Proteins that have been successfully loaded into EVs include luciferases and fluorescent proteins for in vivo and in vitro tracking,^[^ ^93^ ^]^ antibodies for targeting, active enzymes, RNA‐binding proteins,^[^ ^94^ ^]^ and immunogens for vaccines. Silva et al. loaded green fluorescent protein (GFP) into EVs and fused them with different sorting proteins, illustrating that cargo‐loading efficiency depends on the sorting protein, revealing a new range of candidate proteins and enhancing the ability to target receptor cells.^[^ ^79^ ^]^ One study loaded Cas9 ribonucleoprotein (RNP) via electroporation into EVs secreted by hepatic stellate cells (EV‐RNP). EV‐RNP promotes the specific accumulation of RNP in the liver, targeting p53 to upregulate the expression of apoptosis regulator and cyclin E1 (CcnE1).^[^ ^80^ ^]^ This provides a potential means for the EV treatment of liver diseases with precision and tissue specificity. It has also been found that systemic administration of EVs loaded with phage‐identified peptide‐modified dystrophin splice‐corrected phosphorodiamidate morpholino oligomers resulted in an 18‐fold increase in dystrophin in the quadriceps muscle of a mouse model with no detectable significant toxicity.^[^ ^66^ ^]^ This result demonstrates the ability of EV‐anchored peptides to directly and efficiently functionalize and capture EVs, providing delivery tools for the biomedical field. Besides, protein scaffolds have been studied for loading EVs,^[^ ^95^ ^]^ allowing a more efficient loading of various cargoes such as active enzymes, cytokines, antibodies, and ligands, onto the EVs.

### 4.4 EVs‐Mediated Gene Editing Technology

**Figure 4:**
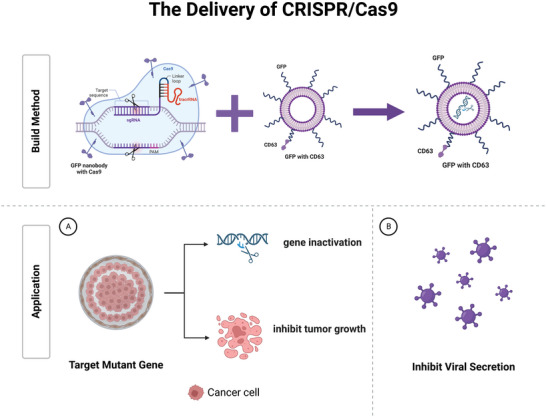
EVs‐mediated CRISPR/Cas9 system. The EVs can be engineered to target mutant genes or virus‐infected cells by fusing CD63 with GFP, while the loaded Cas9 proteins fused to the GFP nanobody. The delivery system can effectively inactivate target genes, inhibit tumor growth, and reduce virus replication rate.

Clustered regularly interspaced short palindromic repeats (CRISPR) associated endonuclease‐9 (Cas‐9), consisting of sgRNA and Cas9 protein, can be used for gene editing, offering great hopes for genetic disorders. Cas9 can be loaded into EVs through inducible protein dimerization, showing promise as a vehicle for delivering CRISPR/Cas9‐based protein systems, such as those required for base, primer, and genomic or epigenomic editing. Osteikoetxea et al. designed a reversible protein dimer system that significantly improved gene editing efficiency.^[^ ^96^ ^]^ Currently, studies have found that CRISPR/Cas9 is inadequate to precisely edit the gene in recipient cells, though EVs deliver them. To address this, a functionalized engineered EV delivery system has been designed by fusing GFP and GFP nanobodies with marker proteins CD63 and Cas9. Therefore, Cas9 protein can be selectively and efficiently captured by loading into EVs due to the affinity of GFP‐GFP nanobody.^[^ ^97^ ^]^ The results show that the new modified EV system abrogates target genes more precisely and efficiently, achieving preliminary success in treating lung adenocarcinoma cell line A549. Besides, editing genes, CRISPR/Cas9 can also be used for the antiviral treatment of the human hepatitis B virus (HBV) and human papilloma virus by transfecting cells with antiviral guide RNAs and Cas9 plasmids. Coculture experiments showed that the cleavage rate of viral DNA and inhibition of HBV replication was approximately 30%. After adding GW4869, an inhibitor of EV secretion, the CRISPR/Cas9‐mediated the HBV replication suppression rate was significantly diminished,^[^ ^81^ ^]^ shedding new light on antiviral therapy. Coincidentally, McAndrews et al. demonstrated that CRISPR/Cas9 is loaded with EVs and delivered to recipient pancreatic cancer cells to target the mutant Kras^G12D^ oncogenic allele, suppressing tumor–cell proliferation and inhibiting tumor growth.^[^ ^82^ ^]^ Besides, CRISPR/Cas9 can be encapsulated into hybrid EVs,^[^ ^98^ ^]^ which are produced by incubating the EVs with liposomes. This approach may combine the advantages of two nanomaterials (**Figure** **4**).

## 5 Therapeutic Applications of EVs as Carriers

### 5.1 Cancer Treatment

**Figure 5:**
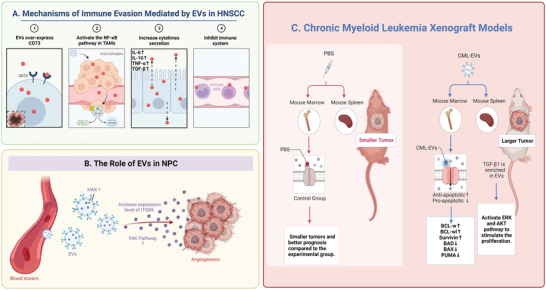
The role of EVs as endogenous carriers in treating A) head and neck squamous cell carcinoma, B) nasopharyngeal carcinoma, and C) chronic myeloid leukemia.

Cancers are considered a massive threat to human health and are the second leading cause of mortality worldwide. Nowadays, some novel treatments based on targeting cancer cells, including anti‐cancer drugs and immunotherapy drugs, have emerged.^[^ ^99^ ^]^ EVs, as excellent nanocarriers, have gained significant attention due to their efficient and selective delivery, offering possibilities for modern drug delivery. Zhu's group designed an EV system with two positive charges that can enter the phospholipid bilayer in a very short time and load both aggregation‐induced emission luminogens (AIEgens) and PPI, combining glutamine inhibition and photodynamic therapies to effectively inhibit tumor growth in a subcutaneous model of gastric cancer.^[^ ^100^ ^]^ In patients with colorectal cancer, microsomal triglyceride transfer protein expression is increased in the plasma–EVs as an inhibitor of ferroptosis. In response to this novel mechanism, EVs loaded with oxaliplatin may reverse resistance to chemotherapy.^[^ ^101^ ^]^ Gu et al. applied hUCMSCs‐EVs to load with triptolide (TPL) by engineering them with a cyclic peptide to overcome the disadvantages like poor solubility and high hepatic and renal toxicity, establishing a bionic‐targeted drug delivery system. This system possessed superior tumor targetability and prolonged the half‐life of TPL, obviously inhibiting tumor growth and extending the survival time of the mouse model of malignant melanoma via the caspase cascade and mitochondrial pathway.^[^ ^102^ ^]^ A disease strongly associated with hepatocellular carcinoma is cirrhosis, and thus cancer‐associated fibroblasts (CAFs) play an important cellular communication function in HCC. Wang et al. found that miR‐335‐5p was downregulated in HCC cells cocultured with CAF. After treatment with EV‐loaded miR‐335‐5p, the target mRNA was downregulated and slowed down the growth and invasion of hepatocellular carcinoma.^[^ ^103^ ^]^ In response to the difficulty of neoantigen identification due to the HCC heterogeneity, Zuo and co‐workers designed a specific vaccine DEX_P&A2&N_ by combining DC‐derived EVs (DEX), function domain of high mobility group nucleosome‐binding protein 1 (HMGN1), immune adjuvants that promote DC recruitment and activation, the HCC‐targeting peptide P47(P) and *α*‐fetoprotein epitope (AFP212‐A2).^[^ ^104^ ^]^ A significant tumor retardation has been found in orthotopic HCC mice after intravenous administration by recruiting and activating cross‐presenting CD103^+^CD11^+^ and CD8*α* ^+^CD11c^+^ CD in the tumor. In head and neck squamous cell carcinoma, EVs expressed CD73 to promote malignant progression and mediate immune evasion by activating the NF‐*κ*B pathway in tumor‐associated macrophages thereby increasing cytokine secretion such as IL‐6, IL‐10, TNF‐*α*, and TGF‐*β*1 to inhibit the immune system (**Figure** **5**A).^[^ ^105^ ^]^ A previous study showed that HAX1‐riched EVs are associated with metastasis in nasopharyngeal carcinoma (NPC). This phenomenon has been explained that HAX1‐riched EVs promote angiogenesis through the activation of the FAK pathway in endothelial cells by increasing the expression levels of ITGB6 (Figure 5B).^[^ ^106^ ^]^ In chronic myeloid leukemia (CML) xenograft models, mice treated with EVs from CML cells had larger tumors than controls treated with PBS.^[^ ^107^ ^]^ Anti‐apoptotic molecules like BCL‐w, BCL‐xl and survivin have been found to increase while pro‐apoptotic molecules like BAD, BAX, and PUMA have been found reduced both in vitro and in vivo samples. Additionally, TGF‐*β*1 was enriched in the EVs of CML cells and can stimulate CML cell proliferation by activating ERK, AKT, and anti‐apoptotic pathways (Figure 5C). EVs function in tumorigenesis, development, cancer immunity and drug resistance. It provides a new target to improve chemotherapeutic efficacy, and offers a novel idea for precise therapy.

### 5.2 Treatment of Cardiovascular Diseases

**Figure 6:**
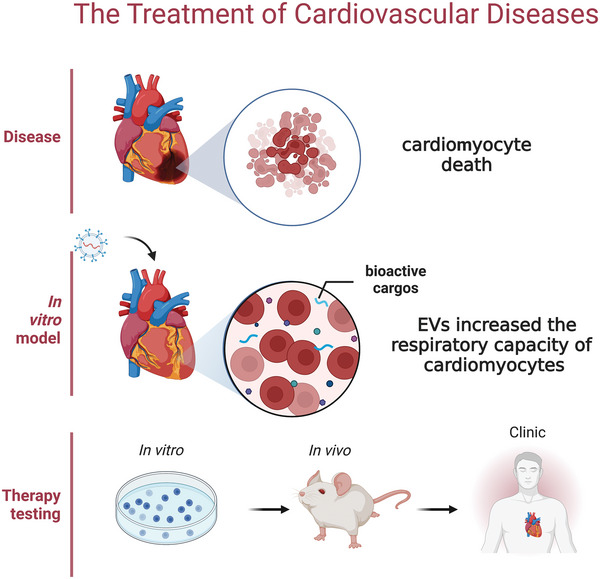
The treatment of cardiovascular diseases. Vascular endothelial EVs increased the respiratory capacity of normoxic cardiomyocytes, and EVs rescued cardiomyocytes exposed to IRI, possibly by providing bioactive cargos to support multiple metabolic.

Cardiovascular diseases (CVDs), including coronary artery diseases, myocardial infarction (MI), heart failure, stroke, and so on, are some of the most common diseases that threaten human health and cause a substantial medical burden. Current pharmacological treatment for CVDs has low bioavailability, low retention, and poor targeting, leading to disappointing efficacy and complicated side effect. Engineered EVs have many superior properties compared with traditional drugs and can overcome their limitations, serving as alternative therapeutic options for CVDs. EVs derived from various stem cells induce cardioprotective effects during ischemia reperfusion injury (IRI). Moran mapped the protein content of human vascular EVs and assessed their specific cardio‐protection, and found that cardiac cell death was alleviated after treatment with EVs during IRI. Besides, they found that vascular endothelial EVs increased the respiratory capacity of normoxic cardiomyocytes, implying that the EVs rescue cardiomyocytes exposed IRI possibly by providing bioactive cargos to support multiple metabolic.^[^ ^108^ ^]^ In the MI of swine, we also observed that the recovery had improved when treated with the EVs from human induced pluripotent stem cells (hiPSCs).^[^ ^109^ ^]^ An abdominal aortic aneurysm (AAA) is a localized expansion of the abdominal aorta whose complication is lethal like the rupture of the aortic wall. Compared with normal aortic walls, the features of an aneurysm, such as inflammatory cell involvement and ECM degradation, especially elastin degradation, can be utilized as potential targets. Based on this, an elastin antibody tethered to nanoparticles to target aneurysms has been designed.^[^ ^110^ ^]^ The drugs specifically target AAA and accumulate in the degraded elastic layer in the mouse model after treatment with the system.^[^ ^111^ ^]^ Thus, it would be possible to use engineered EVs to treat AAA. Although few studies have loaded drugs into EVs to treat cardiovascular disease, relevant studies have used EV mimics for cardiovascular disease. For example, synthetic mesenchymal stem cells, an EV mimic formed by wrapping poly lactic‐co‐glycolic acid particles around MSC cell membranes, deliver growth factors to the damaged heart and promote cardiac regeneration, as well as maintaining therapeutic activity after cryopreservation.^[^ ^112^ ^]^ Targeting the damaged site and increasing the circulation time are the most important functions of the EV mimic (**Figure** **6**).

### 5.3 Treatment of Liver Diseases

For liver cells in a pathological state, cellular stress causes the activation of various signaling pathways, producing various biological effects. Hepatocyte‐derived EVs fuse directly with target hepatocytes and transfer neutral ceramidase and sphingosine kinase 2 (SK2), increasing the synthesis of sphingosine‐1‐phosphate (S1P) in target hepatocytes, thereby mediating liver repair and regeneration.^[^ ^113^ ^]^ Furthermore, intravenously injected EVs show prominent accumulation in the liver and a reduction in rapid renal clearance, making them an appropriate treatment for liver diseases. Studies on hepatitis, liver failure, and liver cancer have all found that EVs play an important role in their treatment. The mice treated with an MSC‐conditioned medium, the main ingredient being EVs, had lower serum INF‐*γ*, IL‐1*β*, and IL‐6 levels and higher serum IL‐10 levels after 48 h in the acute liver failure (ALF) model.^[^ ^114^ ^]^ Furthermore, Zhao et al. showed that the expression levels of pro‐apoptotic proteins Bax and caspase‐3 were reduced, and that of anti‐apoptotic protein Bcl‐2 was improved after EVs derived from bone marrow mesenchymal stem (BMSC) cells treated in ALF mice.^[^ ^115^ ^]^ Therefore, we hypothesized that BMSC‐EVs prevent hepatocyte apoptosis through autophagy in ALF. EVs derived from the livers of Schistosoma japonicum‐infected mice induce neutrophil extracellular traps (NETs) released by delivering miR‐142a‐3p to target WASL and prevent the development of Schistosoma japonicum.^[^ ^116^ ^]^ miR‐142a‐3p and NETs can upregulate CCL2 expression, activating the immune system and recruiting macrophages to inhibit Schistosoma japonicum development. Additionally, WSAL knockout can accelerate the formation of NETs, suggesting that WASL is a potential therapeutic target and that delivering miR‐142a‐3p by EVs can be effective in treating of Schistosoma japonicum. Following more research on EVs, it is believed that the application of EVs in liver diseases will have a higher status in the future.

### 5.4 Treatment of Kidney Diseases

Growing evidence has confirmed a central role of EVs in kidney physiology and pathology.^[^ ^117^ ^]^ EVs in the urine or circulation might participate in regulating kidney functions and the communication between the glomeruli and nephron. Potential biomarkers associated with kidney diseases are detectable in the EVs of urine, such as detecting AQP1 in acute kidney injury to evaluate levels. Evidence of therapeutic use of EVs has been observed. EVs from MSCs have therapeutic properties to accelerate kidney recovery, perhaps because of the abundant expression of C–C motif chemokine receptor‐2 (CCR2), which can bind to ligand CCL2. It has also been proven that CCR high‐expression EVs can reduce the CCL2 concentration and suppress the macrophage activation. Meanwhile, the protective effects will be impaired after CCR2 knockdown in the renal ischemia model.^[^ ^118^ ^]^ Klotho is a single‐pass transmembrane protein in the kidney and is crucial in renal tissue regeneration. EVs derived from urine contain Klotho, Klotho expression is decreased during acute or chronic damage. When ineffective fibroblast EVs are loaded with Klotho, protection is reacquired.^[^ ^17^ ^]^ Additionally, the EVs loaded with Klotho had better reno‐protective effects than the Klotho alone in the AKI mouse model. Tang et al. reported an approach to deliver interleukin‐10 (IL‐10) with EVs by engineering macrophages to relieve AKI. The stability of IL‐10 and the targeting to kidney have been enhanced, showing significant amelioration of renal tubular injury and potentially preventing the transition to chronic kidney disease.^[^ ^119^ ^]^ MSCs‐EVs have been recognized as another promising cell‐free therapy for AKI, a supramolecular hydrogel containing Arg‐Gly‐Asp (RGD) peptide has been developed to augment the MSCs‐EVs efficacy for treating AKI. The data showed that RGD‐EV hydrogels provided superior rescuing effects on renal function, reduced renal tubular damage and promoted cell proliferation in the early stage of AKI by RGD binding with integrin.^[^ ^120^ ^]^ In summary, the capacity of EVs to shuttle cargo to the kidney lead to be utilized for treating kidney disease, particularly as EVs can protect the transported molecules, thus prolonging the half‐life, evading the off‐target effects, and altering cellular processes. However, deeper research remains needed to make the strategy better.

### 5.5 Treatment of the Nervous System Diseases

**Figure 7:**
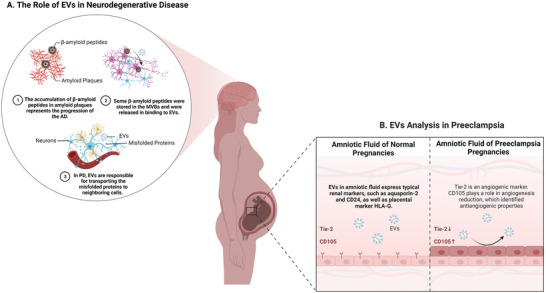
The role of EVs as endogenous carriers in neurodegenerative disease and preeclampsia. A) In Parkinson's disease, EVs are responsible for transporting the misfolded proteins to neighboring cells. In Alzheimer's disease, a fraction of *β*‐amyloid peptides were stored in the multivesicular bodies and released on binding to EVs. EV‐associated protein, Alix, was specifically enriched in amyloid plaques of patients with AD. B) Comparison between amniotic fluid EVs from healthy and preeclampsia pregnancies showed that the surface marker CD105 was especially upregulated, meaning that EVs from amniotic fluid of preeclamptic pregnancies might have the potential property of anti‐angiogenic.

Noteworthy, in the nervous system, EVs promote neuron growth and survival, stimulating tissue repair and regeneration.^[^ ^16^, ^121^ ^]^ In the mice peripheral nervous system, Schwann‐cells‐derived EVs promote nerve axon regeneration both in vitro and in vivo, possibly because of the inhibition of RhoA, a suppressor of axon regeneration. In neurodegenerative diseases, including prion disease, EVs derived from either non‐neuronal or neuronal cells can spread the infection by releasing PrP^Sc^ (an abnormal pathogenic protein), suggesting a novel method of transmission. And it made clinical prion disease when the EVs are inoculated into mice.^[^ ^122^ ^]^ In Parkinson's disease (PD), EVs are responsible for transporting the misfolded proteins to neighboring cells, providing a fresh insight into PD's therapeutics. The consensus that the accumulation of *β*‐amyloid peptides in amyloid plaques represents the progression of Alzheimer's disease (AD) is deeply rooted in the medical community. Research found that a fraction of *β*‐amyloid peptides were stored in the multivesicular bodies (MVBs) and were released on binding to EVs. Further exploration revealed that EV‐associated proteins, called Alix, were specifically enriched in the amyloid plaques of patients with AD (**Figure** **7**A).^[^ ^123^ ^]^ Although it is only an experimental phenomenon, it may play an important role in the field of neurodegenerative diseases. Additionally, EVs loaded siRNA against BACE1 reduced BACE1 associated mRNA and proteins in the mouse's cerebral cortex by 60% and 62%, respectively, and reduced *β*‐amyloid peptides by 55%.

### 5.6 Treatment of the Immune System Disease and Diagnostic Applications

EV involvement is also observed in immune system diseases, in which HIV‐infected dendritic cells release HIV‐1 particles is associated with EVs. Wiley et al. isolated the EVs from 24 h supernatant of HIV‐infected dendritic cells, and compared the infectivity potential with cell‐free virus particles, it was 10‐fold more than.^[^ ^124^ ^]^ It is possible by activating and enhancing virus adhesion or by inducing conformational changes in the viral envelope through EVs. Whatever the mechanism, inhibition of infected EVs could provide a new additional cellular target of antiretroviral therapy. During pregnancy, EVs suppress the immune system of mother, and promote fetal survival by activating the EVs with immunosuppressants.^[^ ^19a^ ^]^ Previous studies have shown^[^ ^125^ ^]^ that EVs from preeclamptic pregnancies promoted endothelial permeability and damaged mice's vasculature and glomeruli. Gebara and co‐workers^[^ ^126^ ^]^ compared amniotic fluid EVs from healthy and preeclampsia pregnancies, found that the surface marker CD105 was especially upregulated, indicating that EVs from amniotic fluid of preeclamptic pregnancies might have the potential property of anti‐angiogenic. It would be significant in the future to evaluate CD105 expression by EVs isolated from the amniotic fluid of pregnancies, as a diagnostic test (Figure 7B). Tears are superior non‐invasive samples. Recently, an incorporated tear‐EV analysis (iTEARS) has been described,^[^ ^127^ ^]^ which is based on negative‐pressure‐oscillation strategy and can isolate EVs with high yield and purity within five minutes. It has identified 904 proteins and demonstrated CALML5, KRT6A and S100P for the classification of dry eye disease, and showed that miR‐145‐5p, miR‐214‐3p and miR218‐5p, are dysregulated during diabetic retinopathy development, suggesting EVs can be further studied as diagnostic markers.

### 5.7 Treatment of COVID‐19

**Figure 8:**
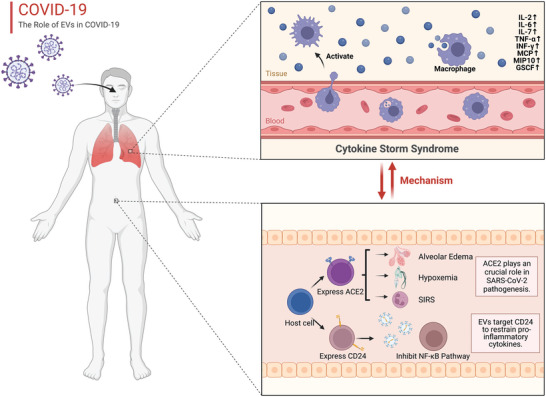
The role of EVs in the mechanism of cytokine storm syndrome and COVID‐19. CSS is an EV‐involved profound inflammatory immune response in which the expression of IL‐2, IL‐6, IL‐7, TNF‐*α*, INF‐*γ*, MCP, MIP 10, and GSCF is upregulated. SARS‐CoV‐2 can induce the secretion of pro‐inflammatory cytokines to trigger alveolar edema, dyspnea, hypoxemia, and systemic inflammatory response syndrome. EVs targeting CD24 inhibit the NF‐*κ*B pathway and restrain them from secreting proinflammatory cytokines, suggesting that CD24 is a potential immune target in COVID‐19.

Since SARS‐CoV‐2 was declared as a pandemic coronavirus disease 2019 (COVID‐19) by WHO, the infection has spread to more than 200 countries and has had 546357444 confirmed cases worldwide and 6336415 mortalities according to the data from the WHO of July 4, 2022. This represents a global threat to public health as it is highly contagious (ranging from 2 to 6 people that one contagious person will infect)^[^ ^128^ ^]^ and has serious sequelae; to date, there is no specific treatment. Evidence from lipidomic and metabolomics analyses of plasma shows that monosialodihexosyl gangliosides (GM3)‐rich EVs are associated with COVID‐19 pathogenesis, and that GM3 levels increase with disease severity.^[^ ^129^ ^]^ A subset of patients with severe COVID‐19 will develop severe “Cytokine Storm Syndrome” (CSS), a profound inflammatory immune response, which threatens multiple organs. SARS‐CoV‐2 can induce the secretion of pro‐inflammatory cytokines to trigger alveolar edema, dyspnea, hypoxemia, and systemic inflammatory response syndrome (**Figure** **8**). The angiotensin‐converting enzyme 2 (ACE2) plays a crucial role in SARS‐CoV‐2 pathogenesis by allowing the virus to enter host cells.^[^ ^130^ ^]^ Nevertheless, CD24 can inhibit the NF‐*κ*B pathway and restrain it from secreting pro‐inflammatory cytokines, suggesting that CD24 is a potential immune target in COVID‐19. In the work of Shapira et al., they devised a novel anti‐inflammatory drug, EXO‐CD24, a combination of EVs and immune checkpoint CD24 to improve the prognosis of diseases accompanied by CSS.^[^ ^131^ ^]^ In a phase Ib/IIa study, 35 patients with a medium to high COVID‐19 severity were recruited and given 10^8^–10^10^ EXO‐CD24 for 5 d, and no adverse events were observed for 443‐575 d. Wang et al. also reported^[^ ^132^ ^]^ that peritoneal M2 macrophage‐derived EVs exhibited superior anti‐inflammatory potential than immobilized cell lines, resolving CSS by reducing TNF‐*α* and IL‐6 thereby effectively attenuating the damage to multiple organs such as lung, liver, and kidney, as well as oxidative stress. Furthermore, M2‐EVs loaded with functional miRNAs and proteins that regulate the complex interaction of genes, simultaneously inhibit multiple key proinflammatory pathways like JAK‐STAT and p38 MAPK. Recently, several clinical trials have indicated that MSC‐EVs are safe and effective for SARS‐CoV‐2‐associated pneumonia. MSC‐EVs treatment can significantly improve patients’ oxygenation and reconstituted immunity in 72 h with no side effect.^[^ ^133^ ^]^ Meanwhile, mRNA lipid nanoparticle (LNP) vaccine of COVID‐19 has been developed, but its intramuscular injection limits pulmonary bioavailability. However, inhalation therapy allows the lungs to achieve an effective drug concentration and has greater patient compliance, which is a good method for localized drug delivery. Pulmonary EVs are excellent candidates for nanoparticles in inhalation therapy. They exhibit superior mRNA and protein distribution compared with commercially standard biological EVs and LNP,^[^ ^134^ ^]^ enhancing pulmonary bioavailability and therapeutic efficacy. Meanwhile, an inhalable COVID‐19 vaccine has been designed, and it is stable at room temperature for three months. The vaccine combined lung‐derived EVs and recombinant SARS‐CoV‐2 receptor‐binding domain. Compared with liposomes, the EV vaccine is more effective in retaining of active ingredients and clearing viruses.^[^ ^135^ ^]^ COVID‐19 and inhaled EV‐therapeutics developed for pulmonary bioavailability and product stability in other respiratory diseases show excellent performance, outperforming synthetic liposomes.^[^ ^136^ ^]^

## 6 New Storage of EVs and Boosting Secretion

Despite the advantages of EVs, their clinical applications face strong challenges due to their unsatisfactory stability and low yield. Normally, the biological function of EVs is lost over time, leading to a loss of therapeutic effect, even when stored at −80 °C.^[^ ^137^ ^]^ Because the active biogenic molecules are usually unstable, the experiment found that after four freeze‐thaw cycles, characteristic proteins of EVs were reduced to undetectable levels, making freezing storage improper. And the ice crystals generated during freezing can also damage the EV film. After storage 30 d, EV‐associated proteins like ALIX, HSP70/90 and TSG101 will be significantly lost.^[^ ^138^ ^]^ Commonly used cryoprotectants such as DMSO and glycerol promote EV lysis or aggregation, so Gorgens et al.^[^ ^139^ ^]^ optimized a HEPES buffer using alginate and albumin, and even after five freeze‒thaw cycles at ‐80 °C storage conditions, the EVs and their contents remained intact. And researchers dispersed EVs in the concentrated hyaluronic acid solution and removed water by evaporation under continuous airflow to obtain solid‐form EVs, called EV‐microneedles, which remained stable after storing for six months,^[^ ^140^ ^]^ making the extension of EV clinical applications like oral capsules possible.

Various factors have been discovered to boost EV secretion to reach the industrial‐scale production, and plentiful approaches have been explored to improve production yield, though they have limitations. The methods can be broadly divided into four main categories: physical signals, molecular interference, environmental factors, and external inducers such as liposomes and nanoparticles. Mechano sensing is the basic pathway for communication in cell–cell and cell‐microenvironment, triggering the response of the cells to mechanical load and then upregulating the signal of secreting EVs. Several studies have tested the influence of mechanical shear stress on the EV secretion rate, demonstrating that the yield of the treatment of low shear rate on human dermal microvascular endothelial cells is at least 100‐fold higher than that of the control.^[^ ^141^ ^]^ Moreover, another study revealed that this process was mediated by yes‐associated protein (YAP), and the addition of YAP‐inhibitor significantly decreased EV secretion.^[^ ^142^ ^]^ Similarly, Wang et al. examined EV secretion from the periodontal ligament (PDL) cells stimulated with cyclic stretch to investigate the impact of mechanical environment on periodontal immune‐inflammatory homeostasis, reporting that PDL cells under cyclic stretch production of 24 h are 30‐fold higher than those of static cultivation.^[^ ^143^ ^]^ Physical signals also include acoustic stimulation, which stimulates the EV production using sound waves, such as high‐frequency ultrasound,^[^ ^144^ ^]^ and electrical stimulation in that low level of electric treatment can activate the intracellular signaling Rho‐GTPase and endocytosis, which participates in EV formation.^[^ ^145^ ^]^ Molecular interference can regulate the production by influencing a link in the EV formation mechanism. The inhibition of lysosomal function prevents endosome maturation, leading to the accumulation of ILVs, and Ortega et al. found that interfering with endo‐lysosomal trafficking enhances the release of bioactive EVs and retains the regenerative bioactivity.^[^ ^146^ ^]^ Currently, the research on 3D culture of EVs has become very popular, it is a method based on the fact that the properties of EVs can be mediated by microenvironments and in vitro conditions. The yield, protein content, cytokine content, and anti‐inflammatory factors were all increased during hMSCs were grown as 3D aggregates under wave motion, which might be through the activation of ESCRT‐dependent and independent pathways.^[^ ^147^ ^]^ Recently, 3D culture MSC‐derived EVs‐hydrogel hybrid microneedle array has made good progress in spinal cord repair.^[^ ^148^ ^]^ Also, the inhibition of glycolysis, oxidative phosphorylation, and adiponectin can stimulate EV secretion by modulating signaling pathways. Environmental factors, including hypoxia, starvation, and hyperglycemia can all boost EV secretion to a greater or lesser extent, low pH is also known as an irritant (Figure 3). Regarding boosting EV secretion, biosecurity is our priority, and we also need to quantify and analyze the production to ensure that there are no safety issues.

The biggest bottleneck in the field of EV therapeutic applications is the large‐scale production of clinical‐grade EVs. To address this problem, researchers have developed several novel approaches, including the optimization of donor cells, the use of bioreactors, and the development of culture medium additives. For example, current research has identified induced pluripotent stem cells (iPSCs) as a potential source for the large‐scale production of EVs.^[^ ^149^ ^]^ They can be generated from a variety of different somatic cells by genetic methods or chemical induction. iPSCs produce EVs with 16‐fold higher yield and 2‐3‐fold higher EV bioactivity than MSC‐EVs, as well as better biocompatibility with target cells and can be genetically engineered to enhance their immune compatibility.^[^ ^150^ ^]^ In addition, Kojima et al.^[^ ^92^ ^]^ designed an EV‐generating booster, EXOtic, which used cotransfection of plasmids from multiple candidate genes that encode for increasing EV production. Through the determination of EV protein and nanoparticle tracking analysis (NTA), it can be confirmed that the yield of EVs is indeed significantly increased. During the EV secretion phase, donor cells are usually incubated in additive‐free medium because human platelet lysate (hPL) and GMP‐grade fetal calf serum (FCS) themselves contain large amounts of EVs and are difficult to separate from EVs produced by the target cells, which simultaneously limits EV secretion from donor cells. In contrast, Lorenzini et al.^[^ ^151^ ^]^ discovered a technique to prepare EV‐free hPL and EV‐free FCS using the tangential flow filtration (TFF) technique, and the additives they prepared were shown to enable EVs from mammalian cells to be considerably more than those produced in the classical way, without significant effects on morphology, size and contents and their biological activity, which provides a new idea for the large‐scale production of EVs. In particular, it was mentioned in the report on large‐scale production of extracellular vesicles “massivEVs” that high‐density suspension culture of donor cells in a large bioreactor may be suitable for large‐scale industrial production.^[^ ^37^ ^]^

Furthermore, while EVs show great potential as drug delivery systems, clinical trials to study the efficacy and safety of EV therapy are progressing, and EV‐based clinical drugs may be approved in the future and thus actually moved to the clinic. We believe that the scale of EV production should be determined by dose, demand, and shelf life, and therefore, the production scale could be evaluated at an early stage to optimize the production process and avoid expensive production costs. In current large‐scale clinical EV production, it is preferable to use stable and safe iPSCs, which will reduce the cost of production and improve the efficiency. Meanwhile, research and development of novel cell culture media additives are one of the most promising and cost‐effective approaches. Basic research on EVs should also be gradually aligned with industrialization to lay the foundation for future EV‐based applications. We believe that the application of these methods in a scientific and rational combination can significantly improve the yield of EVs, but we also need to devote ourselves to the exploration and discovery of novel ways to increase the yield.

## 7 Obstacles and Challenges of EV‐Based Therapies

EVs are reshaping our perspective on life science and public health, however, EV exploration is not supported by current manufacturing methods. Although many EV‐based therapies have been shown to delay disease progression, the issue of EV purity and yield is a huge hurdle to overcome. Repeated injections of concentrated EVs containing therapeutic molecules are needed to achieve the desired effect. The first issue is the purity of EVs. In fact, regardless of which isolation method is used or which loading strategy is implemented, what is delivered is always a heterogeneous population of EVs containing microvesicles, exosomes, apoptotic vesicles, etc. Even due to the different isolation methods, other vesicular structures or cellular fragments may be present, making it difficult to meet the needs of clinical applications. Therefore, efficient and low‐cost purification techniques are one of the main challenges for EV therapies. Currently, microfluidic techniques combined with physical theory demonstrate unique advantages of integration and high throughput,^[^ ^152^ ^]^ but the lack of large‐scale experimental data to apply to more samples is its main drawback. We believe that combining multiple separation and purification methods at present could better compensate for the shortcomings of each method, although it may cause inefficiencies.

Second, because EVs are secreted by living cells, large‐scale production of clinical‐grade EVs remains an important technical challenge to be addressed. To solve this problem, Kojima designed EXOtic to transfect plasmids that enable increased EV production into donor cells, and developed implantable EV‐producing cells for efficient in situ production and delivery, which opens new ideas for the application of EVs. However, this may also face certain problems, such as the half‐life of the donor cells in vivo and how to ensure that the donor cells always produce therapeutic effects in situ, as well as whether the therapeutic molecules contained in the secreted EVs will remain active. At the same time, if the cargos can produce better therapeutic effects without targeting effects, perhaps we can consider how to reduce the metabolism of circulating EVs. CD47 of EVs has been shown to inhibit their recognition and clearance by macrophages and monocytes, so genetically engineering the secreted EVs of donor cells to express more CD47 could theoretically be effective in reducing the circulating EV reduction. Along similar lines, selecting donor cells as those with long growth cycles in vivo or transfecting relevant genes into donor cells may be one of the ways to solve the problem.

With the development of engineering technology, although research on EVs has made some progress, it is still far from the mature use of EV therapy. We could combine the wisdom of researchers from multiple disciplines as much as possible to provide us with new ideas. For example, optics, chemistry, electromagnetism, and physics have played an important role in the development of microfluidic and high‐throughput biosensing technologies. For example, Tayebi et al. developed an efficient microfluidic device for EV quantification and analysis based on the fluid dynamics capture principle,^[^ ^153^ ^]^ providing a fast and accurate analysis for future EV research and applications. We believe that the close integration of different fields will enable the era of EV therapy to come sooner. In the future, how to balance the drug delivery efficiency, targeting ability, molecular activity and economic cost of EV therapeutics are also major issues worthy of research exploration.

## 8 Conclusion and Future Directions

As cell‐derived nanocarriers, the therapeutic potential of EVs can be precisely and effectively improved using functionalized modifications and loading with bioactive molecules. Although the numerous advantages of EVs bring it further attention as a delivery vehicle, an unsatisfactory yield, purity, and loading efficiency still hinder their clinical application. In addition to the difficulties in large production which is required for therapeutic application, the other production processes like isolation, purification and modification need to be standardized to meet the GMP guidelines. The most important factor is that safety is necessary throughout the production process. Another potential alternative treatment option is to load the therapeutic molecules with EV mimics made of lipids and recombinant proteins. The successful clinical translation of EV‐based nanomedicines still has to overcome many rigorous obstacles, for which we suggest possible strategies:

(a) Combining multiomics studies and biosensor development can help to understand the more complex biological functions of EVs.
(b) In the process of EV engineering, the principle of “upstream is in charge of producing more EVs, and downstream of losing less” should be met as much as possible.
(c) Develop standards for better communication and optimization among researchers.
(d) Combining the strengths of various disciplines to accelerate the transformation and implementation of various EV‐based applications.

We believe that in the near future, with the continuous exploration of researchers and the continuous development of biotechnology, EV‐based research will be translated into clinical applications.

## Conflict of Interest

The authors declare no conflict of interest.
